# The hemostatic profile of cold-stored whole blood from non-greyhound and greyhound dogs over 42 days

**DOI:** 10.3389/fvets.2023.1135880

**Published:** 2023-03-02

**Authors:** James L. Cooper, Claire R. Sharp, Corrin J. Boyd, Melissa A. Claus, Gabriel Rossi

**Affiliations:** ^1^School of Veterinary Medicine, Murdoch University, Murdoch, WA, Australia; ^2^Perth Veterinary Specialists, Osborne Park, WA, Australia

**Keywords:** transfusion, platelet, ROTEM^®^ delta, blood bank, transfusion medicine, coagulation

## Abstract

**Objectives:**

To compare the hemostatic characteristics of cold-stored whole blood (CSWB) from non-greyhound dogs (NGD) and greyhound dogs (GD) over 42 days of storage, notably, platelet closure time (PCT) (NGD only), manual platelet count (PLT) (GD only), ellagic acid (INTEM) and tissue factor activated (EXTEM) rotational thromboelastometry, prothrombin (PT) and activated partial thromboplastin time (aPTT), fibrinogen concentration (FIB), and the activities of factors (F) FII, FV, FVII, FVIII, FIX, FX, FXIII antigen (FXIII:Ag), and von Willebrand factor antigen (vWF:Ag).

**Design:**

Whole blood from 10 NGD and 10 GD, was refrigerated in CPD blood bags at 4°C for 42 days. Blood was analyzed before refrigeration (day 0) and at day 1 (d1), 3, 5, 7, 10, 14, 17, 21, 24, 28, 31, 35, 38, and 42. Multivariate linear mixed effects models were created to evaluate coagulation parameters over time and compare NGD and GD. Data are summarized as estimated marginal means with 95% confidence intervals. Significance was set at *P* < 0.05.

**Results:**

The PCT for all NGD CSWB was above the device limit by d7. The PLT for GD CSWB did not change during storage. The mean alpha-angle for INTEM and EXTEM decreased to <50% of baseline at d38 and d31 for NGD, and d31 and d17 for GD CSWB. The mean maximum clot firmness (MCF) for INTEM and EXTEM reduced to <50% of baseline at d42 and d28 for both GD and NGD. PT and aPTT for NGD and GD increased over time. For NGD CSWB, the mean FVIII and vWF:Ag activities decreased to <50% of baseline at d7 and d28, respectively, and FIB reached 0.982 g/dL by d24. For GD CSWB, FVIII, FXIII:Ag and FV activities decreased to <50% of baseline by d3, d38, and d38, respectively, and FIB was 0.982 g/dL at baseline. Alpha-angle and MCF for both INTEM and EXTEM, and activities for FII, FV, FIX, FXIII:Ag were significantly lower, and vWF:Ag was significantly higher overall in GD CSWB compared with NGD. A significant difference in the pattern of change over time was detected between NGD and GD in EXTEM alpha-angle, INTEM and EXTEM MCF, FII, and FVIII activities.

**Conclusions:**

The *in vitro* viscoelastic parameters of GD and NGD CSWB declines over 42 days, but numerous hemostatic parameters (INTEM and EXTEM alpha-angle and MCF, activity of FII, FV, FV, FVII, FIX, FX, FXIII:Ag, vWF:Ag, and FIB) remain within 50% of baseline for more than 14 days. CSWB from GD compared to NGD has reduced hemostatic activity overall, but a similar pattern of decline for most parameters over time.

## Introduction

Fresh and cold-stored whole blood (CSWB) administration for severe traumatic hemorrhage is associated with reduced morbidity, transfusion requirements, and mortality in people (1–7). This is likely due to the early amelioration of trauma-associated coagulopathy, and mitigation of iatrogenic resuscitation-associated coagulopathy. Trauma-associated coagulopathy, a well-recognized cause of mortality in people, has a prevalence of approximately 30–45% in patients presenting with severe traumatic hemorrhage (8). Trauma-associated coagulopathy has also been reported in dogs with a similar prevalence, although study populations are small and heterogenous with less well-defined inclusion criteria (9–12). Whole blood delivers a physiologically balanced product containing red blood cells (RBC), plasma, and platelets to address the components lost and consumed in severe hemorrhage (1, 5, 8). Presently, the storage of whole blood for later transfusion is a regular practice in veterinary medicine, and there is a need for improved understanding of this product's ideal duration of storage and potential applications (13, 14).

CSWB has numerous appealing attributes including the delivery of a physiological balance of blood components with simple preparation and storage, however, little is known about its hemostatic capacity in dogs. Characterization of the hemostatic potential of CSWB is vital to understanding its utility in veterinary practice. Viscoelastic testing provides a global assessment of the kinetics of clot formation and clot strength under low shear conditions, and may better reflect *in vivo* clotting characteristics than traditional plasma-based coagulation assays (15). In people, *in vitro* viscoelastic parameters for clot strength and formation kinetics for CSWB remain within 50% of measurements at collection for 14–35 days (16–23). Similar results have been reported in canine CSWB over 28 days when measured by kaolin activated thromboelastography (TEG) (24). Rotational thromboelastometry (ROTEM) is another commonly used viscoelastic modality in humans and is emerging in veterinary medicine. While mechanistically similar, reported parameters are not identical between TEG and ROTEM. Presently, there is no published data on dogs, and limited data from people on the viscoelastic profile of CSWB evaluated by ROTEM or the activity profiles of key coagulation factors during storage (17, 20–23, 25, 26).

The platelet component may contribute to the hemostatic capacity of CSWB, however, clinical studies evaluating the *in vivo* efficacy of cold-stored platelets are lacking. In one small study, children with hemorrhagic shock who were administered CSWB stored up to 14 days had post-transfusion platelet counts and TEG parameters comparable to children who received traditional warm platelets (27). Numerous studies evaluating the *in vitro* platelet function have shown promising results, however the findings are dependent on the combination of additives, storage conditions, and method of analysis (28). Platelet closure time (PCT) evaluates the formation of a platelet plug under high shear stress conditions, as opposed to methodologies that evaluate platelet function under low shear conditions such as impedance aggregometry. Edwards et al. demonstrated a rapid decline in impedance platelet aggregation of canine CSWB, while the global clot forming capacity, measured by TEG, was maintained for much longer (24). However, the *in vitro* capacity of canine CSWB to form a platelet plug under high shear conditions remains unknown.

Greyhound dogs (GD) are commonly used in blood donor programs, but exhibit numerous poorly described coagulation differences compared with non-greyhound dogs (NGD), and up to 30% of GD experience delayed postoperative hemorrhage (29–32). Greyhounds have lower platelet counts, shorter PCT, and more hypocoagulable TEG parameters compared to NGD (33–35). Other reported differences between GD and NGD include lower von Willebrand factor antigen (vWF:Ag) and coagulation factor X (FX) activities, and lower fibrinogen concentration (FIB) (35, 36). Given their contribution to canine blood banks and the variability in coagulation components both within GD and between GD and NGD, a better understanding of the hemostatic capacity of GD CSWB is critical and has not yet been evaluated in the veterinary literature.

The primary objective of this study was to characterize changes in the *in vitro* viscoelastic properties, prothrombin time (PT) and activated partial thromboplastin time (aPTT), coagulation factor (F) activities (FII, FV, FVII, FVIII, FIX, FX, FXIII antigen [FXIII:Ag], vWF:Ag, FIB, PCT (NGD only), and estimated manual platelet count (PLT) (GD only) of canine whole blood stored at 4°C for 42 days. We hypothesized that for NGD, the PCT would be greater than 300 s by day seven for all bags. For both NGD and GD, we hypothesized that the mean alpha-angle and maximum clot firmness (MCF) would decrease over time but remain within 50% of baseline until day 14 of storage. For both NGD and GD we hypothesized that the PT and aPTT would increase by no more than 50% from baseline over 42 days. Finally, we hypothesized that the 95% confidence interval for all coagulation factor activities would remain above 50% until day 14, and that the 95% confidence interval for FIB would remain above the previously reported reference interval 0.982 g/L until day 14 of storage (37). Our secondary objective was to compare the alpha-angle, MCF, coagulation factor activities and FIB changes between NGD and GD CSWB over the storage period. For this objective, we hypothesized that the alpha-angles would be lower and MCF weaker across timepoints for GD CSWB compared to NGD CSWB, and that coagulation factor activities from GD CSWB would be lower across timepoints compared to NGD CSWB. A tertiary objective was to document any bacterial colonization within the stored CSWB bags at 42 days of storage, and percent hemolysis during storage.

## Methods

### Animals

Twenty healthy volunteer blood donor dogs (10 NGD and 10 GD) were enrolled from the blood bank of The Animal Hospital at Murdoch University. They were deemed healthy based on history and physical examination. To be suitable for blood donation and enrollment in the study, all dogs were more than 25 kg bodyweight, had a relaxed temperament, were receiving a regular prophylactic flea treatment, were up to date with their core vaccines within the last 12 months, and were not receiving a raw meat diet. There was no randomization, and enrollment was discussed and offered to the owners of all blood donor dogs in order of appearance for donation. Owner consent was obtained prior to enrollment. The study protocol was approved by the Murdoch University Animal Ethics Committee (R3111/19).

### Blood collection

Whole blood was collected routinely as per the hospital blood bank protocol. If necessary, dogs received intravenous butorphanol and/or oral trazodone to facilitate a calm collection. The hair over the venipuncture site was clipped and skin aseptically prepared with chlorhexidine and isopropyl alcohol. A standard volume of whole blood (450 mL) was collected into a closed citrate, phosphate, glucose collection bag (Macopharma^®^ whole blood CPD 600 mL triple bag collection unit, Macopharma^®^, Mouvaux, France; 2.63% sodium citrate, 0.33% citric acid). Jugular phlebotomy was performed using a pre-fixed 16 g needle attached to the collection bags by an experienced blood bank nurse or one of the authors. For storage and future sampling, 150 mL of anticoagulated whole blood was separated into a 150 mL plain transfer bag (Terumo^®^ teruflex T-150 transfer bag, Terumo Corporation, Tokyo, Japan) within 15 min of collection, using a closed, sterile tube welder system (Terumo^®^ TSCD^®^-II sterile tube welder, Terumo Corporation, Tokyo, Japan). A needleless sampling port (SWAN-LOCK^®^ Take set, Codan Corporation, Santa Ana, CA, USA) was inserted into one of the spike ports of the transfer bag for all future sampling. The remainder of the blood donation was processed routinely for addition to the blood bank.

### Blood storage and handling

Each 150 mL transfer bag containing whole blood was stored in a dedicated, monitored, blood bank refrigerator (Liebherr LKUv 1610 Laboratory Refrigerator XT-2000i, Sysmex Corporation, Kobe, Japan) set to 4°C. The blood bags were not moved for any purpose other than sampling, and bags were stored upright along the long edge of the bag.

### Blood sampling

Sampling for testing occurred 15 times per bag at day (d) 0, 1, 3, 5, 7, 10, 14, 17, 21, 24, 28, 31, 35, 38, and 42. The storage time was chosen to investigate beyond previous studies demonstrating coagulation factor stability up to 35 days in human CSWB, and 28 days in dog CWSB (22–24). Forty two days was chosen specifically, since RBC containing products may be stored for up to 42 days depending on the additive solution used (38). The sampling time each day varied except for d0 and d1 which were 24 h apart. The blood bags were removed from the refrigerator and gently inverted 10 times prior to sampling. Non-sterile examination gloves were worn for all sampling. The needleless sampling port was swabbed before and after sampling with alcohol (Alcohol Prep Pad, Henry Schein^®^, NY, USA). A 2 mL volume of blood was removed and then reinfused into the bag prior to sampling to ensure thorough mixing of blood between the sampling port lumen and the bag, after which a 10 mL syringe was used to sample 10 mL of blood for analysis that was then divided for testing. Smaller aliquots (1 and 2 mL) were held at room temperature prior to performing viscoelastic testing (ROTEM^®^ *delta*, Tem International GmbH, Munich, Germany) and platelet function assay (PFA-100^TM^, Siemens Healthineers, Erlangen, Germany), respectively. A 6 mL aliquot of whole blood was centrifuged at 4,000 g for 10 min at 4°C (Thermo Scientific^TM^ Cryofuge^TM^ 5500i centrifuge, Thermo Fisher Scientific^TM^, MA, USA) and the plasma was fractionated and stored at −80°C (Thermo Scientific^TM^, TSX60086 Ultra-Low Freezer, Thermo Fisher Scientific^TM^, MA, USA) for later coagulation factor analysis.

### Analysis of primary and secondary hemostasis

The PFA-100^TM^ with collagen and adenosine diphosphate (ADP) cartridges was used to measure PCT up to the device upper limit of 300 s, for NGD CSWB only. The PCT was run in duplicate at each timepoint until the day on which both duplicates exceeded 300 s. The mean of the duplicates was used in the final analysis. Concurrently, 1 mL of whole blood was used for a complete blood count at an external reference laboratory (Vetpath Laboratory Services, Specialist Diagnostic Services, WA, Australia) or using an in-house analyzer (VETSCAN^®^ VSPro Coagulation Analyzer, Abaxis Inc., CA, USA) to confirm a minimum platelet count for accurate interpretation, since less than 100 × 10^9^ platelets per liter can prolong PCT (39). The commercial laboratory reported either an automated count, manual platelet count, or a subjective estimate of “adequate”. An estimated manual platelet count (PLT) was performed for all GD CSWB at all timepoints by assessment of a May-Grunwald Giemsa stained blood smear by a board-certified clinical pathologist. A platelet count was calculated by multiplying the mean number of platelets across 10 oil immersion fields (×100 objective) of the central monolayer by 15 × 10^9^/L (40).

Global coagulation was assessed by the ROTEM^®^ delta system (ROTEM^®^ *delta*, Tem International GmbH, Munich, Germany) assays INTEM and EXTEM. In relation to plasma coagulation factors, INTEM provides an assessment of intrinsic coagulation factor activity by recalcification of the citrated sample with *star-tem* (calcium chloride) and activation of the intrinsic coagulation pathway with *in-tem* reagent (containing rabbit partial thromboplastin phospholipid, and ellagic acid). EXTEM evaluates the extrinsic coagulation factors by *star-tem* recalcification and activation of the extrinsic pathway with *r ex-tem* reagent (containing recombinant tissue factor, phospholipid, and a heparin inhibitor). The ROTEM parameters alpha-angle and MCF were analyzed to evaluate both the rate of *in vitro* clot formation, and maximum clot strength. The PFA-100^TM^ and ROTEM assays were commenced between 30 and 45 min after aliquots were removed from the CSWB transfer bag, as per manufacturer instructions and previously published consensus guidelines (41, 42). Samples from the first timepoint (d0) were run prior to refrigeration.

The ACL TOP^®^ CTS 300 (Werfen, Barcelona, Spain), a multiparameter photo-optical turbidimetric analyzer, was used to measure PT, aPTT and the coagulation factor activities for FII, FV, FVII, FVIII, FIX, FX, FXIII:Ag, vWF:Ag, and FIB. Frozen samples (−80°C) were thawed in a water bath at 38°C and batch analyzed by dog to reduce the impact of inter-assay variation. Immunodepleted human plasma reagents were prepared according to manufacturer guidelines (HemosIL^®^, Instrumentation Laboratory, Werfen, Barcelona, Spain). A modified PT was performed to measure the activity of FII, FV, FVII, and FX, and a modified aPTT was used to assess FVIII and FIX. For these modified PT and aPTT assays, the resultant factor activity is proportional to the correction time for the clotting time of each factor deficient plasma. Fibrinogen concentration was measured by the Clauss method using bovine thrombin. A frozen plasma pool collected from 40 healthy NGD was used as a reference sample to calibrate the individual coagulation factors. This calibration pooled plasma was assigned a factor activity of 100%. Calibration was performed for each new lot number of immunodepleted plasma reagent. Calibration with NGD pooled plasma was used for measurement of both NGD and GD samples. The FIB was calibrated against the manufacturer's citrated human calibration plasma (HemosIL^®^, Instrumentation Laboratory, Werfen, Barcelona, Spain). Daily quality control was performed using the manufacturer's normal control plasma (HemosIL^®^, Instrumentation Laboratory, Werfen, Barcelona, Spain) and coefficients of variation (CV) were reported. All frozen plasma was thawed and analyzed within 12 months of collection.

### Hemolysis

To build on knowledge regarding the red cell storage lesion of canine CSWB, serial percent hemolysis was calculated for both NGD and GD CSWB at d21, d28, d35, and d42. Percent hemolysis was calculated as previously described: % hemolysis = [supernatant hemoglobin (g/L) × (100 – packed cell volume)] / total hemoglobin (g/L) (43). Total hemoglobin concentration (g/L) was measured from a citrated whole blood aliquot (Hemocue^®^ Hb 201+ System, Hemocue^®^, Ängelholm, Sweden) at the same time as ROTEM analysis, and supernatant hemoglobin was measured in citrated plasma (Hemocue^®^ Plasma/Low Hb system, Hemocue^®^, Ängelholm, Sweden) after centrifugation (4,000 g for 10 min at 4°C; Thermo Scientific^TM^ Cryofuge^TM^ 5500i centrifuge, Thermo Fisher Scientific^TM^, MA, USA). Concurrently, a packed cell volume was determined on the citrated whole blood aliquots.

### Bacterial culture

Given the risk of bacterial contamination of CSWB during extended storage, and the potential for this to influence coagulation test results, bacterial culture was performed on d42. Specifically, 4 mL of blood was sampled and aseptically transferred into a pediatric blood culture bottle (BacT/ALERT^®^ Culture Media, Biomérieux, Marcy-l'Étoile, France) for aerobic and anaerobic bacterial culture, after the final sample was removed for coagulation testing. Culture was performed at an external reference laboratory (Vetpath Laboratory Services, Specialist Diagnostic Services, WA, Australia).

### Statistical analysis

Descriptive statistics for PCT are reported as the day number at which all results exceeded the reference interval and analyzer upper limit of detection. The mean PCT for all bags was also compared to the institutional reference interval of NGD for freshly collected citrated (3.2% sodium citrate) whole blood (52–101 s). To evaluate the ROTEM parameters (alpha-angle and MCF), PT, aPTT, coagulation factor activities and FIB over time and compare NGD and GD, multivariate linear mixed effects models were created with fixed effects of time point, dog type (NGD or GD) and interaction between time point and dog type, with a random variance of individual bag nested within dog type. A univariate linear mixed effects model for GD was created to evaluate change over time of PLT. Data are summarized as estimated marginal means with 95% confidence intervals. Assumptions of homoscedasticity and normality of residuals were evaluated by visual assessment of plots of residuals by fitted values and Q-Q plots of residuals, respectively. Significance was set at *P* < 0.05. For coagulation factor activity, the earliest time point at which the lower bound of the 95% confidence interval is below 50% activity is reported for both NGD and GD CSWB. Similarly, the earliest time point at which the lower bound of the 95% confidence interval of FIB is below 0.982 g/L, a previously published reference interval, is reported for both NGD and GD CSWB (37). For PT and aPTT, the earliest time point at which the upper bound of the 95% confidence interval is above a previously published reference interval is reported (37). The reference intervals for FIB, PT, and aPTT in that study were generated from citrated (3.2% sodium citrate) plasma from healthy dogs frozen at −80°C for <1 month (37). A power calculation was not performed due to the novel nature of the study and population. Open-source statistical software [R version 4.1.2 (44), with *nlme* (45) and *emmeans* (46) packages] was used to perform the analysis. For the hemolysis data, the first time point at which the percent hemolysis was >1% is reported. To provide objective information about the magnitude of hemolysis in the bags that exceeded 1%, we have reported the median value (Min–Max) for maximum percent hemolysis.

## Results

### Blood donor population

Donor dogs included 10 NGD of various breeds (4 mixed breed dogs, 2 golden retrievers, and 1 each of a Doberman, Australian shepherd, border collie, and Rhodesian ridgeback), and 10 GD. The median age for NGD was 68 months (range: 36–117 months) and 78 months (range: 33–107 months) for GD. There were 3 females and 7 males in both the NGD and GD groups, and all dogs were neutered. All enrolled dogs except one GD had donated blood more than once prior. Trazodone (Trazodone hydrochloride, Bova Aus, NSW, Australia) was administered orally to 2 NGD (5 and 10 mg/kg, 90 min prior to collection), and 1 GD (5 mg/kg 30 min prior to collection). Butorphanol (Ilium Butorgesic injection, Troy Laboratories Pty Ltd, NSW, Australia) was administered IV to 2 NGD (0.2 mg/kg), and 1 GD (0.15 mg/kg).

### Platelet closure times: Non-greyhound dogs

Platelet closure times were performed on 9 out of 10 bags of NGD CSWB, at 22 timepoints, with concurrent automated platelet counts at 11 of the 22 timepoints. The CV between duplicates was calculable for 16 of 22 timepoints and was >15% for four of those 16 timepoints (NGD dog #2 d0, 22%; NGD dog #3 d0, 22%; NGD dog #5 d1, 22.7%; NGD dog #7 d0, 20%). Platelet counts from all 11 timepoints were greater than 90 × 10^9^/L, or subjectively graded as “adequate platelet number”. One NGD did not have a PCT performed for any timepoint because they were enrolled prior to the addition of this testing to the study design. The mean PCT for all bags was greater than 300 s by d7 of storage, and greater than the institutional reference interval (101 s) by 24 hours of storage.

### Estimated manual platelet count: Greyhound dogs

**Figure 1:**
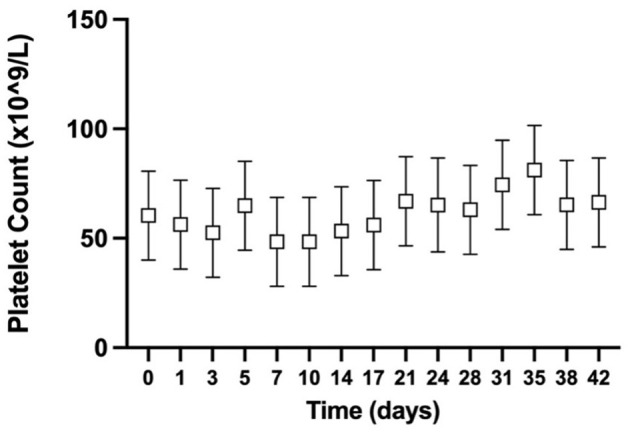
Serial estimated marginal means with 95% confidence intervals of the estimated manual platelet count from greyhound whole blood cold-stored for 42 days.

The estimated manual platelet count was performed for all GD CSWB at all timepoints. There was no statistically significant difference in the PLT across timepoints (*P* = 0.11; Figure 1).

### Prothrombin time and activated partial thromboplastin time

**Figure 2:**
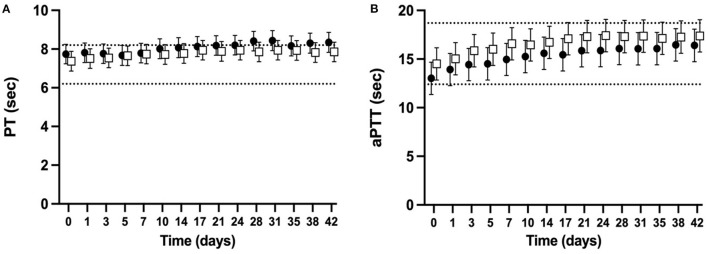
Serial estimated marginal means and 95% confidence intervals for prothrombin time (PT; **A**) and activated partial thromboplastin time (aPTT; **B**) of whole blood from non-greyhound (**•**) and greyhound (□) dogs cold-stored for 42 days. The dotted lines represent the published reference interval for citrated plasma from dogs of a mix of breeds using the ACL-TOP^®^ 300 CTS (37).

The PT and aPTT were measured for 98% of planned measurements. For both NGD and GD CSWB, the mean PT increased over time (*P* < 0.0001) with the upper limit of the confidence interval above a previously published reference interval (37) at d0 for NGD and d7 for GD (Figure 2A). When comparing the PT of NGD and GD CSWB, the model found no significant overall difference when time point was not considered (*P* = 0.2817), and no difference in the pattern of change over time (*P* = 0.8987). For both NGD and GD CSWB, the mean aPTT also increased over time (*P* < 0.0001) (Figure 2B). For NGD CSWB, the lower limit of the confidence interval was below the previously published reference interval until d3, and then the confidence intervals remained within the reference interval for the remainder of storage (37). The confidence intervals for aPTT from GD CSWB were within the reference interval at d0, but the upper confidence interval for GD CSWB crossed the upper reference interval limit at d7 of storage (37). When comparing the aPTT of NGD and GD CSWB, the model found no significant overall difference when time point was not considered (*P* = 0.2327), and there was no difference in the pattern of change over time (*P* = 0.9765).

### Rotational thromboelastometry

**Figure 3:**
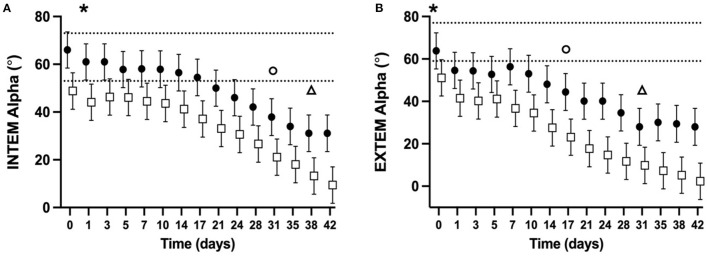
Serial estimated marginal means and 95% confidence intervals for the INTEM **(A)** and EXTEM **(B)** alpha-angle of whole blood (CSWB) from non-greyhound (**•**) and greyhound (□) dogs cold-stored for 42 days. The open triangle (Δ) and open circle (o) denotes when the estimated marginal mean falls below 50% of baseline for non-greyhound and greyhound CSWB, respectively. The dotted lines represent the institutional reference interval for freshly collected non-greyhound dog citrated whole blood. The asterisk (*) indicates when the lower bound of the 95% confidence interval for non-greyhound CSWB falls below the institutional reference interval for citrated whole blood. No greyhound ROTEM reference intervals are available for comparison.

**Figure 4:**
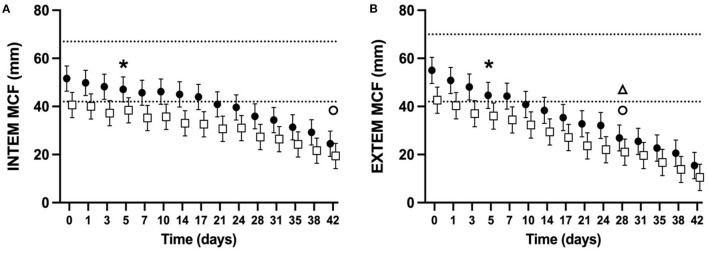
Serial estimated marginal means and 95% confidence intervals for the INTEM **(A)** and EXTEM **(B)** maximum clot firmness (MCF) of whole blood (CSWB) from non-greyhound (**•**) and greyhound (□) dogs cold-stored for 42 days. The open triangle (Δ) and open circle (o) denotes when the estimated marginal mean falls below 50% of baseline for non-greyhound and greyhound CSWB, respectively. The dotted lines represent the institutional reference interval for freshly collected non-greyhound dog citrated whole blood. The asterisk (*) indicates when the lower bound of the 95% confidence interval for non-greyhound CSWB falls below the institutional reference interval for citrated whole blood. No greyhound ROTEM reference intervals are available for comparison.

ROTEM parameters were determined for 98% of planned measurements. Some ROTEM data was missing for two NGD (NGD #8 d10, d14, and d17, NGD #9 d42) and one GD (GD #9 d31, d35, d38). The mean alpha-angle for INTEM (Figure 3A) and EXTEM (Figure 3B) assays for both NGD and GD declined over time (*P* < 0.0001 for INTEM and EXTEM). The mean INTEM and EXTEM alpha-angle reached 50% of baseline at d38 and d31, respectively, for NGD CSWB, and at d31 and d17, respectively, for GD CSWB. The mean MCF for INTEM (Figure 4A) and EXTEM (Figure 4B) assays for both NGD and GD declined over time (*P* < 0.0001 for INTEM and EXTEM). For NGD CSWB the mean INTEM MCF did not reach 50% of baseline for 42 days of storage but for GD CSWB 50% of baseline was reached at d42. For both NGD and GD CSWB the mean EXTEM MCF reached 50% of baseline at d28. There was a significant overall difference between dog type for INTEM alpha-angle (*P* = 0.0025), EXTEM alpha-angle (*P* = 0.0007), INTEM MCF (*P* = 0.0109) and EXTEM MCF (*P* = 0.0222). A significant difference between dog type in the pattern of change over time was identified for EXTEM alpha-angle (*P* = 0.0195), INTEM MCF (*P* = 0.0167), EXTEM MCF (*P* = 0.0039), but not INTEM alpha-angle (*P* = 0.5920).

### Coagulation factor activity

Coagulation factor activities were measured for 97.3% of planned measurements. Data for all coagulation factors is missing at some time points from two NGD (NGD #8 d42, and NGD #9 d5, d10, d17) and one GD (dog #9 d31, d35, d38). The CV for control results were < 15% for all factors except FVII (15.8%), vWF:Ag (15.9%), and FXIII:Ag (48.3%). The high CV for FXIII:Ag was due to a large increase in control value results after a change in reagent lot that did not decrease from this point onwards despite additional changes in reagent lot. This change occurred following the complete analysis of the first four NGD and two GD.

**Figure 5:**
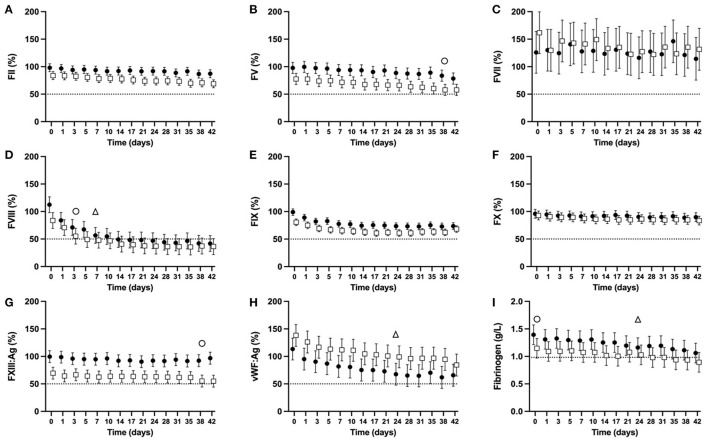
Serial estimated marginal means and 95% confidence intervals for percentage coagulation factor activities [FII **(A)**, FV **(B)**, FVII **(C)**, FVIII **(D)**, FIX **(E)**, FX **(F)**, FXIII:Ag **(G)**, vWF:Ag **(H)**], and fibrinogen concentration **(I)** of plasma derived from whole blood from non-greyhound (**•**) and greyhound (□) dogs cold-stored for 42 days (CSWB). Plasma was separated from CSWB and frozen at −80°C before thawing and batch analysis of coagulation factor activity. The open triangle (Δ) and open circle (o) denotes when the estimated marginal mean falls below 50% of baseline for non-greyhound and greyhound CSWB, respectively. The dotted line represents 50% coagulation activity **(A–H)**, and a fibrinogen concentration of 0.982 g/L **(I)**.

There was a significant overall change over time regardless of dog type in the activities of FII, FV, FVIII, FIX, FX, vWF:Ag (*p* < 0.0001 for all, Figures 5A, B, D–F, H), FXIII:Ag (*P* = 0.0341, Figure 5G), and FIB concentration (*p* < 0.0001, Figure 5I). There was no significant change over time for FVII activity (*P* = 0.6863, Figure 5C).

For NGD CSWB, the lower bound of the 95% confidence interval fell below 50% activity for FVIII at d7 (Figure 5D) and for vWF:Ag at d24 (Figure 5H). The FIB reached 0.982 g/L at d24 of storage, then increased slightly and did not fall below 0.982 g/L until d35 (Figure 5I). The lower bound of the 95% confidence intervals for activities of FII, FV, FVII, FIX, FX, and FXIII:Ag in NGD CSWB all remained >50% for 42 days of storage (Figures 5A, B, D–G).

For GD CSWB, the lower bound of the 95% confidence interval fell below 50% activity for FVIII by d3 (Figure 5D), FV by d38 (Figure 5B), and FXIII:Ag by d38 (Figure 5G). The FIB for GD CSWB was below 0.982 g/L at d0 of storage and continued to decline thereafter (Figure 5I). The lower bound of the 95% confidence intervals for activities of FII, FVII, FIX, FX and vWF:Ag in GD CSWB all remained >50% for 42 days of storage (Figures 5A, B, D–F, H).

When coagulation factor activity was compared between NGD and GD CSWB, an overall difference was statistically significant for FII (*P* = 0.0025), FV (*P* = 0.0018), FXIII:Ag (*P* = 0.0001), FIX (*P* = 0.0015), and vWF:Ag (*P* = 0.0391) activities (Figure 5). Coagulation factor activities for GD compared to NGD CSWB were overall lower for FII, FV, FIX, and FXIII:Ag, and higher for vWF:Ag. A difference in the pattern of change over time was only statistically significant for FII (*P* = 0.0283) and FVIII (P = 0.015) activities (Figures 5A, D), where GD CSWB exhibited a steeper decline in the serial estimated marginal means over time relative to NGD CSWB. The models comparing NGD and GD CSWB found no significant overall difference for FVII (*P* = 0.6707), FVIII (*P* = 0.22), FX (*P* = 0.3488), or FIB (*P* = 0.0934), and no difference in the pattern of change over time for FV (*P* = 0.4728), FVII (*P* = 0.6894), FIX (P = 0.0747), FX (*P* = 0.3022), FXIII:Ag (*P* = 0.5724), vWF:Ag (*P* = 0.7885) activities, or FIB (*P* = 0.4014).

### Hemolysis

Hemolysis data were available for 9 out of 10 NGD CSWB bags. The number of samples available at each time point were as follows, d21 (*n* = 7), d28 (*n* = 7), d35 (*n* = 7), d42 (*n* = 6). Additionally, two NGD had hemolysis measurements performed on d31 and d38 (rather than d28 and d35). Five NGD had < 1% hemolysis at all time points up to and including d35 (NGD #2) and d42 (NGD #5, 6, 8, 9). The four remaining NGD exceeded 1% hemolysis for the first time on day 28 (NGD #7, 10), and day 31 (NGD # 3, 4). For the four NGD with > 1% hemolysis the median value of maximum percent hemolysis was 1.85% (Min 1.74, Max 2.15).

Hemolysis data was available for 10 out of 10 GD CSWB bags. The number of samples available at each time point was as follows d21 (*n* = 9), d28 (*n* = 10), d35 (*n* = 10), d42 (*n* = 8). Five GD had < 1% hemolysis at all points up to and including d42 (NGD #5-9). The five remaining GD exceeded 1% hemolysis for the first time on day 21 (NGD #10), day 28 (NGD #3), and day 35 (NGD #1, 2, 4). For the GD with > 1% hemolysis the median value of maximum percent hemolysis was 1.49% (Min 1.34, Max 2.51).

### Bacterial cultures

Aerobic and anaerobic cultures were performed for 19 out of 20 CSWB bags at d42. For one NGD CSWB bag there was inadequate remaining blood at d42 to submit for culture. Two had positive aerobic cultures; one NGD bag had a light pure growth of *Microbacterium oxydans*, and one GD bag had a light pure growth of *Staphylococcus capitis*. Both isolates were pan-susceptible to all antimicrobials tested.

## Discussion

The results of our study demonstrated that, as hypothesized, CSWB from NGD and GD retains numerous clot-forming characteristics well beyond 14 days despite a rapid loss of platelet function under high shear stress conditions. When measured by ROTEM, CSWB from GD exhibited slower clot formation kinetics and weaker clot strength compared with NGD, a difference that was not detected by traditional plasma-based assays PT and aPTT. There was no significant difference in the pattern of change over time for the measured viscoelastic parameters from NGD and GD CSWB. Numerous differences in coagulation factor activities were also identified.

As hypothesized, the PCT for all bags of NGD CSWB reached the upper limit of detection within 7 days of storage, and was greater than the institutional reference interval within 24 h of storage. A rapid loss in platelet function measured *in vitro* is a well-described limitation of both cold and room-temperature platelet storage techniques in people (47) and dogs (48). The PFA-100^TM^ evaluates platelet function under high shear stress, and best represents the *in vivo* high flow, high pressure conditions of the arterial circulation. Achieving a normal PCT requires an adequate number of platelets with adequate expression and function of surface receptors, and degranulation of the procoagulant contents of the alpha and dense granules. Surface receptors of particular importance are those active during primary hemostasis such as expression of the vWF, collagen and selectin binding proteins glycoprotein GPVI, GP1b-IX-V complex, integrin α_IIb_β_3_, integrin α_2_β_1_ and the ADP receptors P2Y_1_ and P2Y_12_ (39, 49). Our data does not allow for a precise understanding of the rapid loss in platelet function in NGD CSWB, but mechanisms involving vWF, collagen and ADP binding are hypothesized to contribute (39, 42). Our results contrast one study evaluating PCT of human CSWB where the PCT was still able to be measured for all bags between 10 and 14 days of storage (26). However, the results of the referenced study are difficult to interpret, because before 10 days of storage, numerous bags did not generate a PCT, and numerous flow obstruction errors were reported. Consistent with our data, a study of six human CSWB units over 28 days demonstrated rapid and progressive PCT prolongation, with the PCT exceeding the healthy adult reference interval within 24 h of storage (50). While the PFA-100^TM^ has seldom been used for evaluation of stored blood products, the results of our study suggest that there is a loss in the ability of platelets from NGD CSWB to form strong aggregations to resist high flow, and the *in vitro* loss of this function occurs much earlier than the decline in viscoelastic clot strength. The *in vivo* significance of this early prolongation of PCT is unknown but may indicate that the ability of platelets from CSWB to contribute to strong clots in high shear conditions is lost within the first few days of storage. Edwards et al. investigated platelet function under low shear stress by impedance aggregometry using ADP and collagen agonists in NGD CSWB (24). They similarly identified a faster decline in isolated platelet function relative to decline in TEG parameters, and similar findings have also been reported in human CSWB (16, 50, 51). One reason for this disproportionate early decline in isolated platelet function using these assays is the absence of the strong platelet agonist thrombin, which is generated in large amounts in exogenously activated ROTEM and TEG. Due to financial limitations of our study, PCT was not performed on the GD CSWB in lieu of additional coagulation factor assays for both groups of dogs. Given the rapid prolongation of PCT in NGD CSWB and low PLT recorded for GD CSWB we would expect similar PCT results in the GD, particularly given data from people demonstrating the inverse relationship between the sample platelet count and PCT, especially below 100 × 10^9^ platelets/L (52, 53). Given this recognized limitation, the PFA-100^TM^ is likely to be an inaccurate tool for assessment of isolated platelet function in GD CSWB. While no data exists for PCT in GD CSWB, one study reported a shorter mean PCT in healthy GD relative to NGD, however only 20% of the NGD data points were outside of the range of GD in that study, and there was a difference in the upper limit of the standard deviation of only 26 s (33). Whether this would be the same for PCT in GD CSWB over time is unknown.

Consistent with our hypothesis, the PLT for GD CSWB remained stable throughout storage. The CSWB PLT range for GD in our study was lower than published automated and manual platelet reference intervals for healthy greyhounds (35, 54) suggesting that some platelet lysis or clumping may have occurred during the initial collection period but did not continue during storage. Additionally, citrate dilution likely also made a small contribution to the lower platelet counts. Similar stability in the platelet count of NGD CSWB was reported by Edwards et al. (24). In people, the serial platelet counts of CSWB are more variable, but all appear to show an early rapid decline in the first few days of storage before a plateau for the remainder of storage (16, 19, 21, 22, 25, 50). Our data also aligns with others, in that the platelet count of canine and human CSWB alone does not provide a good indication of *in vitro* platelet contribution to clot formation.

As hypothesized, both the PT and aPTT from NGD and GD CSWB showed a similar pattern of change over time, with no significant overall difference in means during storage. Furthermore, PT and aPTT did not increase to 50% above baseline in either NGD or GD for the duration of storage. The overall change in both PT and aPTT was minimal and of similar magnitude to that reported for another NGD CSWB cohort (24). Interestingly, FII, FV, and FIX activities were all lower in GD than NGD overall, which would contribute to varying degrees of prolongation in aPTT. Our data suggests that the magnitude of difference in these coagulation factor activities between dog type was not enough to result in a statistically significant difference in aPTT. However, it should be noted that the contribution of factors XI and XII are unknown because they were not measured in our study and would also affect the aPTT. The PT and aPTT assays did not illustrate a similar magnitude in change to our ROTEM data, likely due to the absence of a cellular contribution to clot formation, and if used as a sole measurement of coagulation of CSWB, may underestimate changes in hemostatic capacity during storage. While the confidence intervals for PT (NGD and GD) and aPTT (NGD only) fell outside a previously published reference interval (Figure 2), the clinical significance of this is unclear due to potential differences in population signalment, differences in the anticoagulation of samples and the intrinsic limitation of PT and aPTT to assess global hemostatic capacity (37).

Supporting our hypothesis, the ROTEM alpha-angle, representing clot formation rate, did not fall below 50% of its baseline measurement in either NGD or GD before d14 of storage (Figure 3). Determinants of the alpha-angle include coagulation factor activity and the resultant rate of thrombin generation, platelet number and function, fibrinogen concentration, and fibrin polymerization rate (55, 56). Our data demonstrate that there is no change over time in platelet number, or the activity of FVII. As such, the change over time in alpha-angle observed on INTEM and EXTEM for NGD and GD is likely due to decreases in platelet function, activities of FII, FV, FVIII, FIX, and X, or fibrinogen concentration. A similar reduction over time in the kaolin activated TEG alpha-angle from NGD CSWB was reported by Edwards et al. (24), and is also observed in viscoelastic studies of human CSWB (16, 17, 19–21, 51).

The alpha-angle for GD CSWB was significantly lower overall across timepoints for both INTEM and EXTEM compared with NGD CSWB. This is consistent with reported TEG alpha values reported from citrated blood samples from healthy GD and NGD (34). Our statistical models suggest that this was likely driven by overall lower activities of FII, FV, and FIX in GD compared to NGD. A difference in the pattern of change over time between dog type was also identified for the EXTEM alpha-angle (Figure 3B). This is likely due, at least in part to the difference in the pattern of change over time in FII and FVIII activities between NGD and GD CSWB. Additionally, unmeasured differences in platelet number or function may have contributed to the EXTEM alpha-angle differences.

The MCF of both NGD and GD CSWB steadily declined over the duration of storage (Figure 4). As hypothesized MCF did not fall to 50% of its baseline measurement before d14 of storage. Fibrinogen concentration, FXIII activity, platelet number, and to a lesser extent platelet function are considered the main procoagulant determinant factors of the MCF in both people and dogs (55, 57–59). The culmination of our data and that of Edwards et al. suggests that the decline in MCF of CSWB during storage is likely due to a decline in platelet function as demonstrated in NGD (24), as well as decreases in FIB, and the activity of FXIII:Ag demonstrated both in NGD and GD in our study. In people, one study using ROTEM also demonstrated a decline in EXTEM MCF from CPD anticoagulated CSWB over time, reaching a statistically significant difference from baseline by d14, however unlike canine CSWB in our study, the median EXTEM MCF only dropped by 12% after 25 days of storage (17). Despite the declines in platelet function and coagulation factor activity during storage, NGD and GD CSWB are still able to maintain *in vitro* clot strength within 50% of pre-storage MCF measurements for at least 42 days (INTEM) and 28 days (EXTEM). The differences in EXTEM MCF changes between people and canine CSWB may be due to inherent interspecies differences or differences in collection, preparation, and storage techniques.

Greyhound dog CSWB had a lower MCF overall across timepoints in both INTEM and EXTEM compared to NGD GSWB. This is consistent with TEG maximum amplitude values reported from citrated blood samples from healthy GD compared with NGD (34). Our data suggests that significant differences in FXIII:Ag (Figure 5G) likely contributed to the lower overall MCF across timepoints. While our model did not find a statistically significant overall difference in FIB, visually there does appear to be considerable dichotomization between NGD and GD CSWB FIB (Figure 5I) and the lack of statistical significance may represent a type II statistical error due to the small sample size. This is plausible since it has been recognized that the reference interval for fibrinogen concentration is lower in healthy GD relative to NGD (35). It should also be noted, that although the lower bound of the 95% confidence interval for FIB from GD CSWB began below the lower bound of a previously published reference interval (37), the proportion of GD and NGD in that study was not reported, and the reference interval is likely not appropriate for GD. A GD-only reference interval for citrate anticoagulated FIB using the ACL TOP has not been published. Although our study did not compare platelet number and platelet function between NGD and GD CSWB, differences in these parameters likely also contributed to the difference in MCF, particularly since the GD CSWB PLT in our study was substantially lower than that from previously reported NGD CSWB (24). Comparison of isolated platelet function of CSWB between dog types has not been reported.

For GD CSWB, the pattern of change in MCF over time compared with NGD CSWB was also significantly different. Our data are unable to explain this as our model did not identify a difference in the pattern of change over time between dog type in either FXIII:Ag or FIB. Other possibilities include differences in the pattern of change over time in platelet number, platelet function, or other unmeasured contributors to the strength of the fibrin meshwork.

As hypothesized, most coagulation factors from both NGD and GD remained above 50% activity for the duration of storage. Only FVIII activity fell below 50% before d14 of storage. While canine and human FVIII activity in cold-stored plasma typically declines earlier than other coagulation factors (60–63), it declined much earlier in our study compared to an investigation of never-frozen liquid platelet-poor canine plasma, where 50% activity was not reached until d35 of storage (64) and was not reached at all by the end of the storage period (14 days) in another study (65). However, our data is consistent with declines in FVIII activity in human CSWB (21, 22, 25). The difference in FVIII activity preservation between cold-stored platelet rich blood products and platelet-poor plasma has also been reported in healthy human citrated blood samples, where FVIII activity is significantly lower after 6 h of storage on ice as whole blood and platelet-rich plasma, compared with platelet-poor plasma (66). The same is true for samples refrigerated at 4°C for 3 h as whole blood prior to centrifugation and FVIII activity measurements in another study (67). Results from these studies suggest that this time-dependent, cold-induced, reduction in FVIII activity is dependent on the presence of platelets. Concurrent measurements of vWF:Ag and electrophoretic analysis of vWF multimers in von Willebrand's disease patients suggest that this process is also dependent on the presence of high molecular weight vWF (66). Previous authors have hypothesized that cold-induced clustering of GP1bα (68) results in increased binding efficiency of vWF to GP1bα, secondarily reducing the activity of FVIII due to its existence as a complex with vWF (66, 67). While it is unknown if this mechanism occurs in dogs, this would be one explanation for the early decline in FVIII activities in our population compared with previous platelet-poor refrigerated plasma products. Due to this rapid decline in FVIII, CSWB appears unsuitable for a patient with a FVIII deficiency (hemophilia A) beyond 5 days of storage for NGD CSWB and 24 h for GD CSWB. However, given the stability of all other factors, it appears suitable for most other indications for hemostatic transfusion in dogs. Interestingly, and again consistent with previous human studies (66, 67), vWF:Ag in our study reached 50% activity 11 days earlier than that from a study of platelet-poor canine plasma (64).

Our study identified that the activities for FII, FV, FIX, FXIII:Ag were all lower in GD CSWB compared with NGD across timepoints. A previous study evaluated GD coagulation factor activities from frozen plasma, that had been sampled, fractionated, and frozen, from room temperature stored, CPD anticoagulated blood, every 8 h for 24 h (36). Contrary to our findings, FX activity was significantly lower in GD plasma compared to NGD in that study, and there were no significant differences in the median activities of FII, FV, or FIX between dog types, when all three timepoints were combined. In our study, FIB appeared visually lower in GD CSWB at d0 and all timepoints compared to NGD, although was not found to be statistically significant. Lower FIB has been reported in healthy GD compared with NGD in two previous studies (37, 42), however no difference was found in another study (39). This variation in results between studies may represent genetic differences in the sample populations, or differences in assay sensitivities and methodologies. Since GD have significantly higher red cell indices than NGD (35), the relative amount of citrate compared to coagulation factors with a standard volume blood donation is also higher, and this dilutional effect also likely contributes to lower coagulation factor activities. Further investigation into the effect of various citrate dilutions in GD CSWB is warranted. Interestingly the activity of vWF:Ag activity in GD CSWB was higher across all timepoints than in NGD, and never fell below 50% activity. This finding is contrary to a previous report where vWF:Ag was significantly lower in GD plasma compared to NGD plasma (36). While the reasons for differences in vWF:Ag activity between dog type is unclear, one hypothesis is that higher numbers of circulating large vWF multimers may be an adaptive evolutionary response to a lower platelet count. The reverse has been demonstrated in people with essential thrombocythemia (69). Inherent difference in the genetic pool of our donor dogs compared to other studies is also possible. The higher vWF:Ag in GD CSWB is particularly interesting given the significantly more rapid decline in FVIII activity in GD CSWB compared with NGD (Figure 5D). Given their relationship as a complex in plasma, this disproportionate decline may indicate a more rapid dissolution of the FVIII/vWF:Ag complex in GD CSWB increasing the opportunity for proteolytic degradation and decline in FVIII activity.

The origins of the bacteria cultured from two bags of CSWB were likely contaminants during the frequent sampling that occurred. *S. capitis* is a well-recognized human skin commensal bacterium, however the origins of *M. oxydans* are less clear, and its status as skin commensal of people or animals, or an environmental bacterium remains contested (70, 71). Due to the single sampling timepoint (d42) for culture, it is unknown at what point during storage bag contamination occurred or if the bag became contaminated at all as opposed to contamination of the sample removed for culture. However, it is unlikely that the contamination affected the precision of our coagulation assays. Additionally, it is unknown if this contamination would occur in CSWB that was not subject to frequent sampling.

Transfusion guidelines recommend that hemolysis of RBC units be checked prior to administration to a patient, and only administered if < 1% hemolysis (72). Although most human and veterinary literature regarding percent hemolysis focuses on packed RBC, it is likely that excessive hemolysis in CSWB may also contribute to transfusion reactions or degradation in hemostatic proteins. Limited studies have assessed hemolysis in human CSWB; one study demonstrated high concentrations of plasma free hemoglobin, (50) while another documented <0.8% hemolysis with CPDA-1 anticoagulant (19). Our study demonstrated marked bag to bag variation. Fifty percent of the CSWB in this study had <1% hemolysis for the duration of storage, but for the bags that exceeded 1% hemolysis, there was marked variation in both the time point at which hemolysis was >1%, and the magnitude of maximum percent hemolysis. As such, hemolysis should be checked prior to administration of CSWB to inform clinician decision making regarding safe administration. Further investigation into the red cell storage lesion in CSWB is warranted, including whether the use of different anticoagulants, or the addition of further RBC preservatives, as occurs for packed RBC storage, may prevent hemolysis exceeding 1% in CSWB. Although additional RBC preservatives, such as saline, adenine, glucose, and mannitol (SAGM), may enhance erythrocyte viability, their effects on the hemostatic capacity of CSWB would require further investigation.

Our study has some limitations. Firstly, this was an *in vitro* study, such that the *in vivo* coagulation effects in a canine recipient of CSWB is unknown. Clinical *in vivo* studies to evaluate the hemostatic effects of canine CSWB are required to understand the utility of this product in a natural population. The study was performed on a small cohort of dogs, and some data were missing, introducing the possibility of type II statistical error. Furthermore, the signalment diversity in our study population may not be representative of all blood donor populations globally, potentially limiting external validity. The PCT was not performed for GD CSWB due to financial limitations, however the rapid prolongation of PCT for NGD CSWB and low PLT (<100 × 10^9^/L) recorded for GD CSWB suggest that PCT would likely have limited utility for evaluation of GD CSWB. While no data exists for PCT on GD CSWB, based on the results of one study, we hypothesize that platelet function under high shear stress from GD CSWB is unlikely to have been better than that from NGD (33). It is also possible the PCT results in our study may not accurately represent the platelet composition of the entire bag of CSWB that would be infused into the recipient. Studies investigating the PCT of recipients of CSWB before and after transfusion would help better understand its *in vivo* effect. In our study, the blood bag was sampled very frequently, and it is possible that the handling and sampling of the blood may have influenced hemostatic performance. Frequent handling, creation of turbulence in the bag, and potential exposure to air during sampling, may have influenced platelet activation and stability of coagulation factors. We did not analyze any bags that were untouched for 42 days to see if the magnitude of change was different. Nonetheless, regular mixing of RBC products is recommended during storage, with frequency varying from every other day to weekly, and thus bag handling in our study likely mimicked clinical blood banking conditions, with the exception of sampling (38). Finally, the ACL TOP^®^ has only undergone analytical validation for PT, aPTT, fibrinogen (Clauss method) and D-dimers in dogs (37). Coagulation factor activity data for canine FV, FVII, FVIII, FIX, FX, vWF:Ag has been previously reported for this device, but has not undergone analytical validation (64, 73). Additionally, the activity of canine FII and FXIII:Ag measured using the ACL TOP^®^ have not previously been reported in dogs. Given the CV of control results for FII were less than 15%, we are confident about its precision, however the accuracy of these measurements is unknown. Due to the dramatic change in the CV of control results for FXIII:Ag associated with a change in reagent batch, the precision of this factor is less reliable, and requires further investigation.

## Conclusion

Our study showed that the *in vitro* rate of clot formation and clot strength of CSWB from NGD and GD as measured by ROTEM decreases over time. This occurs despite a stable PLT (GD only) throughout storage, and a rapid loss of platelet function under high shear conditions within 7 days of storage (NGD only). Nonetheless, all ROTEM parameters and most coagulation factor activities remained within 50% of baseline for more than 14 days, supporting the potential use of CSWB as a hemostatic transfusion product during the first 14 days of storage. However, CSWB from GD compared to NGD appears to have less hemostatic activity but with a similar pattern of decline over time, driven by a lower PLT, and lower activities in FII, FV, FVIII, FIX, FXIII:Ag, and FIB. The *in vivo* significance of these findings remains unknown, but clinicians should consider both the donor's breed and the recipient's transfusion requirements, when selecting CSWB as a hemostatic transfusion product.

## Data availability statement

The raw data supporting the conclusions of this article will be made available by the authors, without undue reservation.

## Ethics statement

The animal study was reviewed and approved by Murdoch University Animal Ethics Committee. Written informed consent was obtained from the owners for the participation of their animals in this study.

## Author contributions

JC contributed to study design, sample collection, laboratory analysis, and wrote the manuscript. CS contributed to study design, sample collection, laboratory analysis, and revision of the manuscript. CB and GR contributed to study design, laboratory analysis, and revision of the manuscript. MC contributed to study design and revision of the manuscript. All authors contributed to the article and approved the submitted version.
